# A 10-year recurrence-free natural course of high-risk stage II cutaneous melanoma: A case report

**DOI:** 10.1016/j.jpra.2025.11.029

**Published:** 2025-11-21

**Authors:** Benedikt Bauer, Marcel Mayrhofer, Maximilian Zarfl, Lars-Peter Kamolz, Hanna Luze, Sebastian Philipp Nischwitz

**Affiliations:** a Division of Plastic, Reconstructive, and Aesthetic Surgery, Department of Surgery, Medical University Graz; b Department of Dermatology, Medical University Graz, Graz, Austria; c Research Unit for Responsible Aesthetics, Division of Plastic, Reconstructive, and Aesthetic Surgery, Department of Surgery, Medical University of Graz, Graz, Austria

**Keywords:** High-risk melanoma, Surgical oncology, Reconstructive surgery, Disease-free survival, Metastasis, Facial reconstruction, Empathy

## Abstract

**Background:**

pT4b melanoma of the skin, especially the nodular subtype with present ulceration, typically entails a poor prognosis with high risk for metastatic spread. The accuracy of classic risk prediction models lacks at times. When situated in the face, these malignancies can pose a specific challenge to reconstructive surgeons. Profound fears of cancer treatment further complicate the management of these cases.

**Case presentation:**

We present a case of facial reconstruction in a 58-year-old female patient with a high-risk stage II melanoma with recurrence-free survival (RFS) despite an almost 10-year delay of surgical treatment due to existential fears.

**Conclusions:**

The presented case provides several key takeaways. First, from a dermatological standpoint, clinicians must expect the unexpected and should look for explanations. Second, from a reconstructive view, complex cases are rarely linear—staged revisions, and setbacks are integral to surgical success. Finally, from a human perspective, sincere empathy, and emotional intelligence are indispensable.

## Background

pT4b melanoma of the skin, especially the nodular subtype with ulceration, typically entails a poor prognosis with high risk for metastatic spread. When located in the face, these malignancies pose a specific challenge to reconstructive surgeons.

We present a case of facial reconstruction in a 58-year-old female patient with high-risk stage II melanoma and recurrence-free survival (RFS) despite an almost 10-year delay of surgical treatment.

## Case presentation

**Figure 1:**
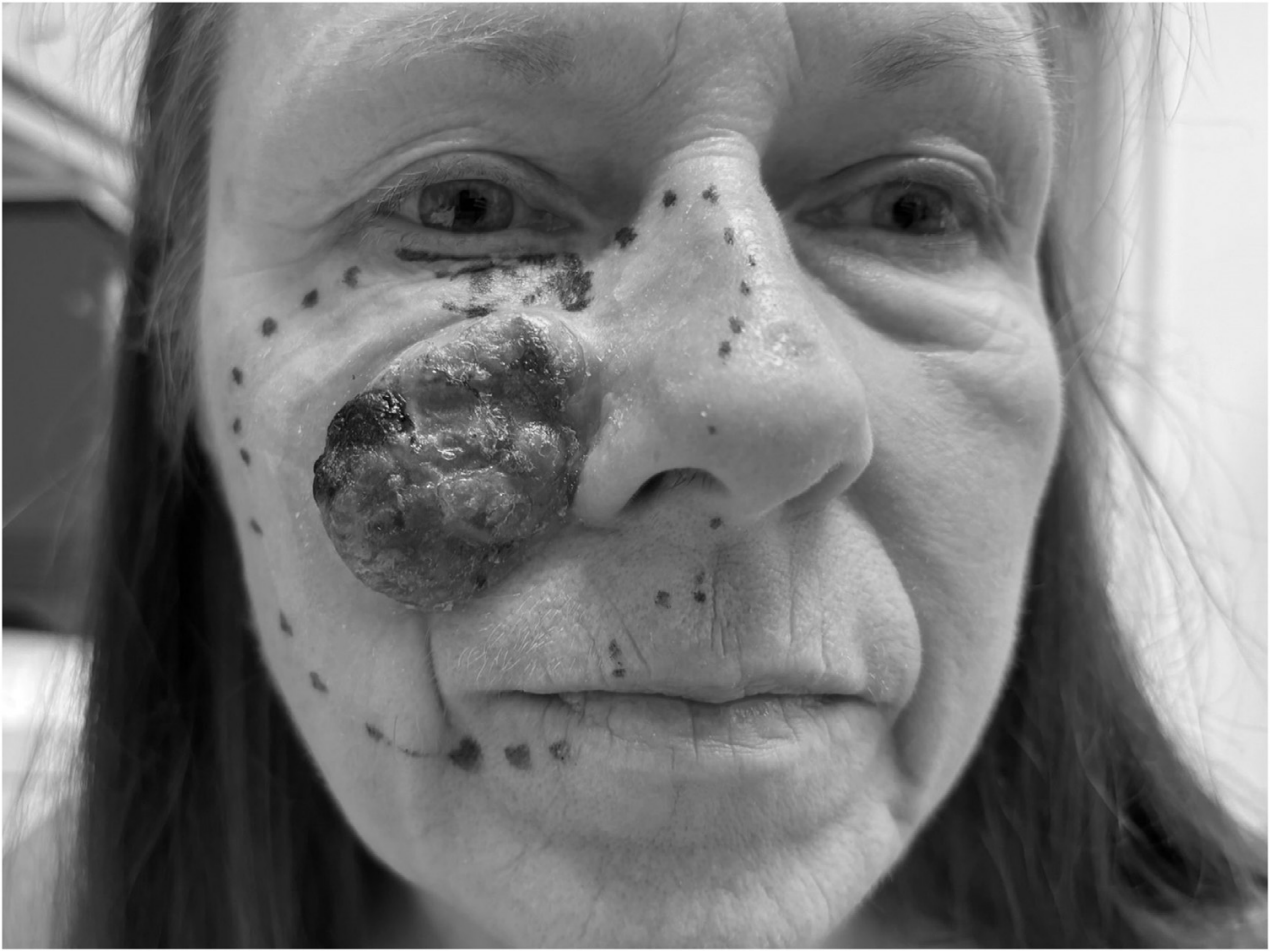
Exulcerated melanoma of the right cheek, nose, and maxillary region. Preoperative markings for resection including parts of the nose, lower eye lid, and perioral region.

The patient first noted a pigmented lesion on the right cheek in 2009, which was excised in 2014. Histological diagnosis revealed a lentigo maligna, though resection margins were unclear. Despite repeated recommendations for re-resection, she delayed treatment until 2022, when she re-presented with a firm, bluish 2.5 cm tumor on the right cheek (Figure 1). Biopsy revealed an ulcerated malignant melanoma, at least stage IIB, BRAF V600 wild type. Comprehensive staging—including MRI, CT of cranium, neck, thorax, abdomen, pelvis, and lymph node sonography—showed no evidence of locoregional or distant metastases.

After rescheduling surgery 13 times, in April 2024 she confided her profound fear of “losing her voice” due to surgery. Addressing this fear built the trust needed to proceed.

**Figure 2:**
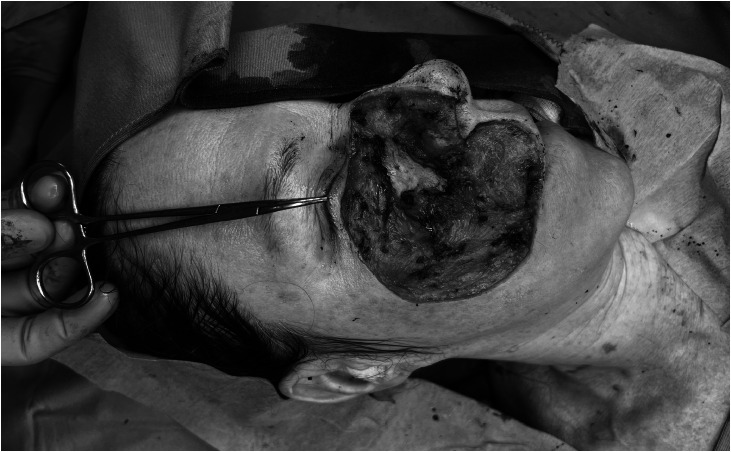
Large defect after tumor resection. Exposed maxilla, partly resected orbicularis orbis muscle, soft tissue defect including the cheek, lower eye lid, nose, and perioral region.

Wide local excision with safety margins adjusted to the surrounding vital anatomy was performed as follows: laterally (cheek) 2 cm, medially (nose) 1.8 cm, cranially (tarsus) 1.2 cm and caudally (upper lip) 1.7 cm. Also, sentinel lymph node biopsy was undertaken. Owing to infiltration of all soft tissue layers, resection included parts of the maxillary periosteum, most of the lower eyelid, angular artery, infraorbital nerve, facial nerve branches, parts of the orbicularis oris, levator labii, levator labii alaeque nasi muscles, and superficial soft tissue from philtrum, upper lip, and nose (Figure 2). The defect was temporarily closed with Epigard.

**Figure 3:**
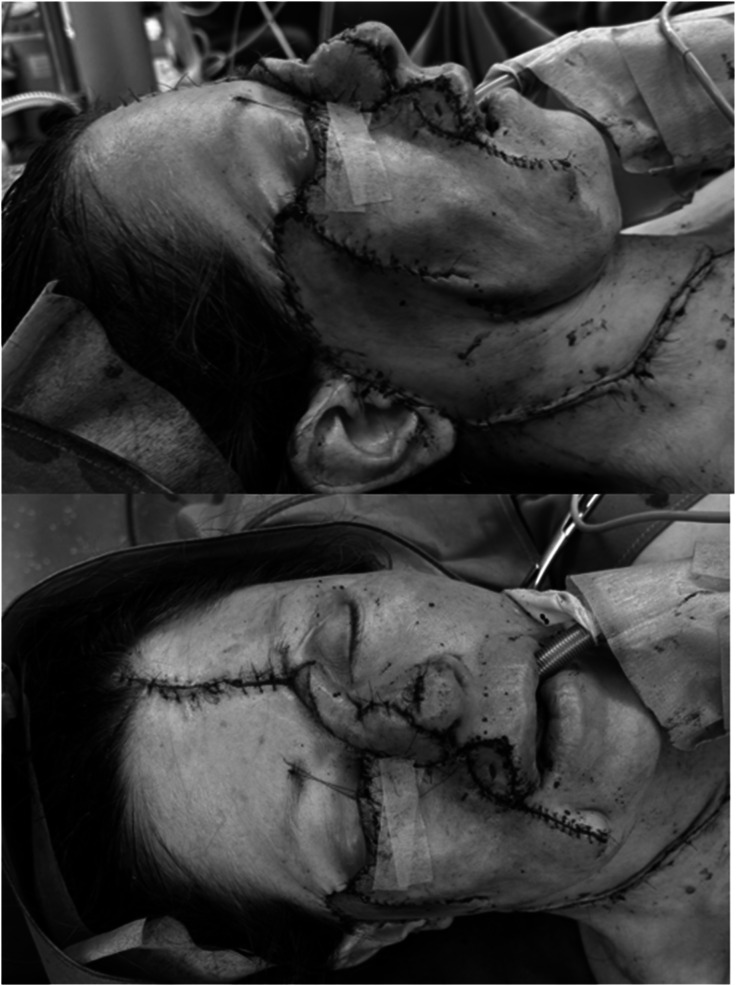
Intraoperative photograph after first step of facial reconstruction: cervicofacial and paramedian forehead flaps harvested and transposed into the defect.

Histopathology confirmed an ulcerated nodular malignant melanoma, R0, Breslow thickness beyond the measurable limit of 14 mm, pT4b, with negative sentinel node (AJCC 2017: pT4b pN0 (0/4sn) cM0, Stage IIC). Following R0 resection, multi-step reconstruction posed a challenge due to the extent and involvement of several aesthetic units of the face. A cervicofacial flap was raised, dissected in the subcutaneous plane transitioning to the subplatysmal plane to preserve facial and cervical structures. To restore nasal contour, a contralateral paramedian forehead flap was elevated. At the nasal tip, an additional lesion (10 × 7 mm) was excised and closed primarily (Figure 3). Here, histopathology showed an in-situ squamous cell carcinoma reaching the lateral margin. Re-excision (now R0) was performed and the defect closed primarily.

Due to partial flap necrosis, debridement and repositioning of the cervicofacial flap were required in a third surgery. After stable healing, residual defects were covered with full-thickness skin grafts from the contralateral supraclavicular groove. Ectropion of the lower eyelid, caused by subtotal excision of orbicularis oculi and scar contraction, was corrected by tarsal strip canthoplasty.

**Figure 4:**
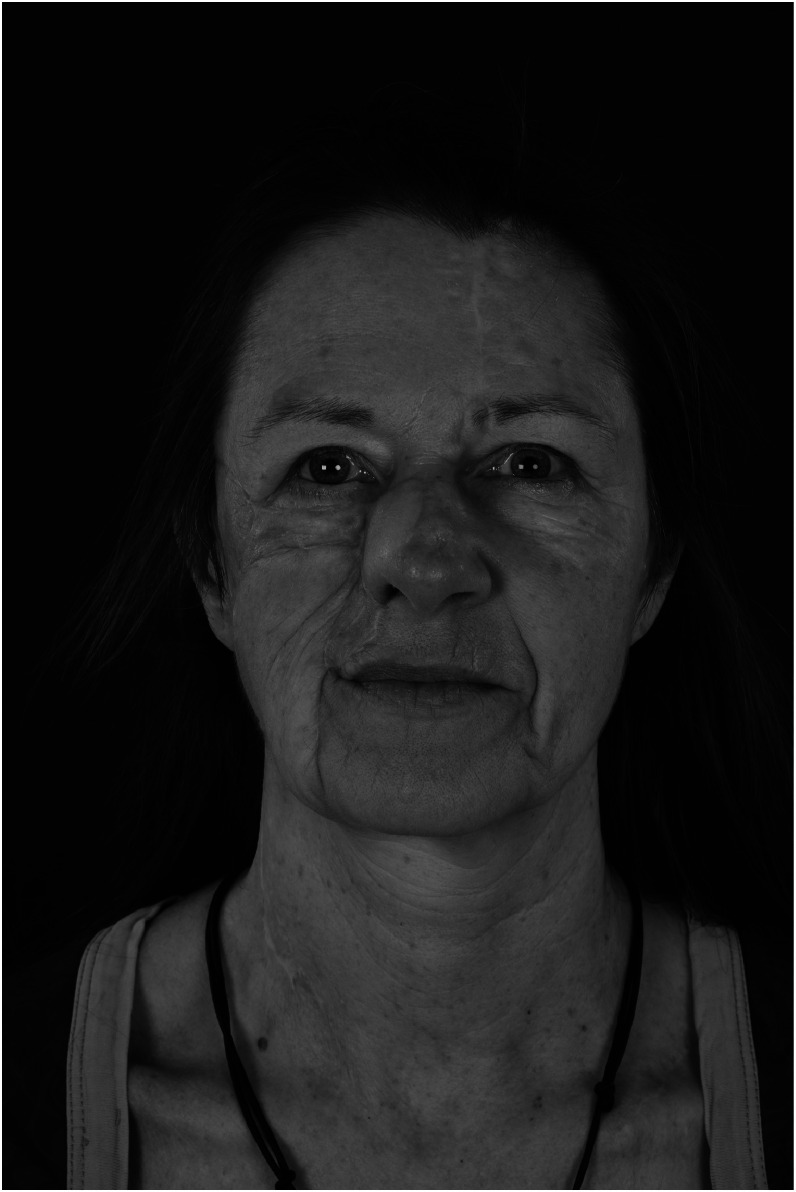
Preliminary result after wound healing. Not “perfect,” but socially unobtrusive and emotionally accepted. Further surgery for scar correction was proposed to and postponed twice by the patient.

Three months after initial reconstruction, the paramedian forehead flap was detached and thinned. This delay resulted from uncertainty regarding flap perfusion in the absence of circumferentially healthy skin. Despite further refinement being proposed, the patient postponed twice and remains undecided. The current result is functionally sufficient, socially unobtrusive, and emotionally accepted (Figure 4).

Dermatologic follow-up every 3 months including whole body inspection for melanotic and amelanotic lesions as well as serial imaging and laboratory monitoring confirmed ongoing remission. As of June 2025, the patient remains disease-free, having continuously declined adjuvant therapy despite multiple attempts of clarifying indication, benefits and risks.

## Conclusion

The presented melanoma is considered a high-risk stage II melanoma according to the American Joint Committee on Cancer (AJCC, eighth edition). These melanomas often show even more aggressive growth and poorer prognosis than stage IIIA and sometimes IIIB melanomas. Reported 5- and 10-year melanoma-specific survival (MSS) rates are 82 % and 75 %, respectively.^1^ The presence of two different entities of skin cancer suggests a high exposure to sunlight in the patient's past, underscoring the importance of protection against sun exposure and frequent dermatologic follow-ups.

Psychological factors such as fear of treatment, cancer-related depression, and existential distress can lead to delayed presentation and medical intervention.^2^ In this case, profound fear hindered necessary treatment for over 10 years, underlining the importance of trust and empathy in the therapeutic relationship.

When comparing adjuvant-treated to untreated stage IIC melanoma, immunotherapy shows clear benefits. In the KEYNOTE-716 trial, 36-month RFS for stage IIC was 71.4 % with pembrolizumab versus 58.0 % with placebo (HR 0.65; 95 % CI), while distant metastasis-free survival (DMFS) was 80.9 % versus 68.1 %.^3^^,^^4^ Similarly, the CheckMate 76 K trial reported a 12-month RFS of 72.0 % (HR 0.51; 95 % CI) for stage IIC patients.^5^

Garbe et al. analyzed two independent melanoma cohorts, reporting 10-year RFS rates for stage IIC of 33.3 % and 49.3 %, and corresponding MSS rates of 70 % and 57.6 %.^6^ These appear less favorable than the IMDDP cohort, which reported 5- and 10-year MSS rates of 82 % and 75 %.^1^

The AJCC 8th edition TNM staging, based on Breslow thickness, ulceration, lymph node, and metastasis status, may fail to predict outcomes reliably, as biological tumor characteristics such as oncogenes and microenvironments are not considered.

Nearly 50 % of all cutaneous melanoma patients harbor a BRAF mutation, more frequent in melanomas of the trunk than those of the head and neck. The most common is the BRAF V600 mutation, accounting for 65 % of all BRAF mutations. Non-V600 mutations occur in 11 % and BRAF fusions in 3 %–6 % of cutaneous melanomas. Other oncogenes involved include NRAS, KIT, TERT, and NF1. Hereditary melanomas represent about 10 %.^7^

A 2020 meta-analysis of 52 studies reported a significantly lower overall survival (OS) (HR 1.23; 95 % CI) in BRAF-mutated compared to wild-type melanoma,^8^ like the melanoma of our patient.

In recent years, the tumor microenvironment has gained attention. Cancer-associated fibroblasts (CAFs) promote tumor progression, metastasis, and therapy resistance by releasing cytokines, chemokines, and growth factors.^9^ In our case the tumor microenvironment was not assessed; hence we can only speculate about its influence on dissemination behaviour of individual melanomas.

Further research is needed on melanoma biology and its impact on prognosis and treatment, as too little is known to date.

The presented case provides three key takeaways. First, from a dermatological standpoint, clinicians must expect the unexpected. Second, from a reconstructive view, complex cases are rarely linear—staged revisions and setbacks are part of surgical success. Finally, from a human perspective, empathy and emotional intelligence are indispensable. By addressing patients’ fears and fostering trust, crucial differences can be made in helping them accept their disease and treatment.

Only by walking alongside our patients can complex oncological and reconstructive challenges be overcome and a real difference made.

## Ethics approval

Ethical approval was not required for this single-patient case report in accordance with the institutional and national guidelines.

## Consent for publication

Written informed consent for publication of this case report and any accompanying images was obtained from the patient.

## Availability of data and material

All relevant clinical information is included within the manuscript. Additional data are available from the corresponding author upon reasonable request. The authors adhered to the STROBE guidelines.

## Funding

No external funding was received for this work.

## Authors’ contributions

BB collected clinical data and drafted the manuscript with the help of MM. MZ contributed to dermatologic assessment and data interpretation. LPK, HL, and SN supervised the surgical aspect and provided help and critical revisions to the manuscript. All authors read and approved the final manuscript.

## Declaration of generative AI and AI-assisted technologies in the manuscript preparation process

During the preparation of this work, the authors used OpenAI´s ChatGPT-5 large language model as well as Grammarly for Windows in order to provide tailored support for tasks such as content organization and improving language and readability. After using these tools, the corresponding author reviewed and edited the content as needed and takes full responsibility for the content of the published article.

## Declaration of competing interest

The authors declare that they have no competing interests.
